# The prevalence and determinant factors of substance use among the youth in Ethiopia: A multilevel analysis of Ethiopian Demographic and Health Survey

**DOI:** 10.3389/fpsyt.2023.1096863

**Published:** 2023-03-23

**Authors:** Tilahun Kassew, Gebrekidan Ewnetu Tarekegn, Tesfa Sewunet Alamneh, Selam Fisiha Kassa, Bikis Liyew, Bewuketu Terefe

**Affiliations:** ^1^Department of Psychiatry, College of Medicine and Health Sciences, University of Gondar, Gondar, Ethiopia; ^2^Department of Epidemiology and Biostatistics, Institute of Public Health, College of Medicine and Health Sciences, University of Gondar, Gondar, Ethiopia; ^3^Department of Pediatrics, School of Nursing, College of Medicine and Health Sciences, University of Gondar, Gondar, Ethiopia; ^4^Department of Emergency Medicine and Critical Care Nursing, School of Nursing, College of Medicine and Health Sciences, University of Gondar, Gondar, Ethiopia; ^5^Department of Community Health Nursing, School of Nursing, College of Medicine and Health Sciences, University of Gondar, Gondar, Ethiopia

**Keywords:** alcohol use, psychoactive substances use, substance use, youth, Ethiopia

## Abstract

**Background:**

In Ethiopia, the youth are more exposed to substances such as alcohol, Khat, and tobacco than other populations. Despite the seriousness of the situation, low- and middle-income nations, particularly Ethiopia, have intervention gaps. Service providers must be made more aware of relevant evidence to combat these problems. This research focused on finding out how common substance abuse is among teenagers and the factors that influence it.

**Methods:**

The 2016 Ethiopian Demographic and Health Survey data were used for secondary data analysis. This survey includes all young people aged 15 to 24 years. The total sample size was 10,594 people. Due to the hierarchical nature of the survey data, a multilevel logistic regression model was employed to uncover the individual- and community-level characteristics related to substances.

**Results:**

In Ethiopia, the overall current prevalence of occasional or daily substance use 30 days prior to the survey was 46.74%. Of the participants, 36.34, 12.56, and 0.95% were drinking alcohol, chewing Khat, and smoking cigarettes/any tobacco products, respectively. Male sex, 20–24 years of age, exposure to media, having a job, and living in large central and metropolitan regions were the factors associated with the problem.

**Conclusion:**

According to the 2016 EDHS, substance use among young people is widespread in Ethiopia. To lower the prevalence of substance use among youth, policymakers must increase the implementation of official rules, such as restricting alcohol, Khat, and tobacco product marketing to minors, prohibiting smoking in public places, and banning mass-media alcohol advertising. Specific interventions targeting at-risk populations, such as youth, are mainly required in prominent central and metropolitan locations.

## Introduction

Substance abuse has become a significant public health issue due to its widespread prevalence across all socioeconomic groups. It has a broad detrimental influence on socioeconomic development and severely endangers public health (1–3). According to a worldwide addiction report in 2017, 1 in 20 to 1 in 5 people aged 15 years and above highly use alcohol, tobacco, and illicit drugs daily (4). Youth includes late adolescents and young adults aged 15 to 24 years (5, 6) who experience substantial changes in numerous facets of life throughout their youthhood, such as rapid physical growth and cognitive, moral, and emotional developments (7, 8). If not properly managed, the youth are prone to risk-taking behaviors, including substance use (9, 10). In the general population, late adolescence and youth are the vital phases at which substance use starts and reaches a peak (11, 12). Alcohol, cigarettes, and cannabis have remained the most regularly consumed substances among youngsters worldwide (13, 14). In Ethiopia, adolescents are particularly vulnerable to substances such as alcohol, Khat, and tobacco products (15–17). While alcohol is the most commonly used and abused substance, tobacco has the highest fatality rate (4). Shisha is another psychoactive substance commonly used in Shashemene town of southern Ethiopia. Shisha is smoking heated, specially prepared tobacco through a pipe. Shisha is also called a water pipe or a Hubble bubble. Like cigarettes, shisha can contain nicotine psychoactive ingredients.

As a result of increased sexual activity, youth who abuse substances are at a higher risk of unintentional injury and death (e.g., automobile accidents and suicide), overdose, and sexually transmitted infections (18–20). The influence of substances alters the mental state of people, increasing the chances of driving accidents and sexually transmitted diseases. Alcoholism and car accidents are well-studied risk factors for injuries and deaths, and substance-induced driving impairment is of increasing concern in many countries around the globe (21). Approximately 54% of sexually transmitted diseases and their associated consequences on Ethiopian patients with HIV/AIDS have been shown to be related to substance use (22). Long-term use increases the risk of several medical illnesses, such as lung disease, heart disease, liver disease, cancer, and psychological issues, such as anxiety, depression, bipolar and psychotic disorders, suicide, and violence (19, 23, 24). Substance abuse has a substantial financial impact due to lost production, deaths, and healthcare costs (13). Furthermore, substance addiction exposes individuals to polysubstance abuse and negatively impacts their quality of life in various ways, including their physical, psychological, social, and environmental activities (25–27).

According to different literature, the prevalence of substance abuse among adolescents varies depending on the substance. For example, people who use substances such as alcohol, Khat, and tobacco ranged from 11.3 to 60%, 9.7 to 74%, and 2 to 56.5%, respectively (16, 17, 28). The combined prevalence of regular or occasional alcohol consumption among youth in eastern Africa was 52 and 15%, respectively (29). In Ethiopia, a systematic review found that youth had a much greater rate of substance use, including alcohol, Khat, and cigarette products, than the general population (19). Another study among high school and university students found that 52.5% have used some substance at some point in their lives, with alcohol accounting for 46.2%, Khat for 24.7%, and smoking cigarettes for 14.7% (30). This demonstrates a disparity across several geographical contexts and time eras. Furthermore, a recent study of university students in Ethiopia found that drinking alcohol, smoking tobacco products, and chewing Khat was attributed to 26.65, 6.83, and 13.13% of youth, respectively (31).

Although substance abuse is a frequent problem among young people, evidence suggests that various factors contribute to its prevalence. Young adults (18–24 years), male sex, living in a divorced/separated family, urban location, unemployment, drug availability, and being out of school are some of the characteristics that increase the chances of substance use among those population groups in low- and middle-income nations (32–35). The sociodemographic factors linked with high substance use are also marital status, religion, higher educational achievement (college and university education), and high income (36–38). Other risk factors for juvenile substance use were peer pressure, having a family member who uses substances, and residing in large cities and regions (16, 32, 34, 35, 39). People also use substances for a variety of reasons, including pleasure, coping with life's challenges, stress and depression relief, staying alert while reading, and improving performance, a lack of alternative forms of recreation in their living environment, high income, and academic dissatisfaction (16, 28, 40). In addition, substance advertising and promotion using mass media are other important factors that engage youth to initiate substance use (41).

Previous literature has focused on substance use among youth attending different educational levels (high school, preparatory school, and college/university students). It lacks focus on community-based education using the national-level population dataset. According to the 2018 WHO Global Alcohol Status Report, Ethiopia does not have a coherent written national policy or action plan on alcohol control (42). Later in 2019, the General of the Ethiopian Food and Drug Authority approved the proclamations to reduce alcohol consumption (43). These include legislation prohibiting the promotion of alcohol on broadcast media and banning smoking in all indoor businesses, public areas, and public transit. The measure also establishes an age limit for alcohol consumption, making it illegal to sell any alcoholic beverage to anybody under 21 years. The Ethiopian government banned the marketing and chewing of Khat to minors in August 2019, comparable to other drugs, to combat Khat addiction (44). Ethiopia's parliament has also passed one of Africa's most burdensome anti-tobacco legislation to address substance abuse's health, social, and economic consequences (41, 45). Even if the laws have been established, the prohibition of alcohol and other substances lacks in preventing harmful consumption among those aged 15 to 24 years. The current study is different from the previous review and single studies assessing the prevalence of substance use among the youth population group at the community level.

This study was based on the 2016 EDHS data that includes the entire nation with a large representative sample size. In addition, this study employed a multilevel logistic model to accommodate the hierarchical nature of the EDHS data. The result of this study will be used to deliver vital health information, which is crucial for policymakers to evaluate programs, design interventions, and strengthen the application of existing policies to reduce the risky consumption of substances among individuals aged 15–24 years. Therefore, this study aimed to assess the prevalence and identify the individual- and community-level determinants of substance use among youth in Ethiopia through a multilevel analysis.

### Hypothesis

- Is substance use high among Ethiopian youth (aged 15–24 years)?
- Are the individual- and community-level factors the determinant factors of substance use in the youth?

## Materials and methods

### Study setting, participants, and procedures

The study was conducted in Ethiopia. Ethiopia is classified into nine regional states, two administrative cities, 611 Woredas (districts), and 15,000 Kebeles. Administratively, each region is divided into zones and zones into Woredas, which is the third administrative division of Ethiopia. Finally, the fourth level, Woredas, is further subdivided into Kebele, the lowest administrative unit. In 2020, Ethiopia's population is estimated to be 114,963,588 people according to UN data (2). The youth aged 15–24 years occupied 19.47% of the total population. Of them, nearly half of the youth are female. The study population was the youth aged 15 to 24 years, who show dramatic changes in multiple aspects of their life.

The data was extracted from the 2016 EDHS data collected between 18 January 2016 and 27 June 2016. The survey collects information on the demographic and health indicators of all household members, with particular emphasis on maternal and child health issues. The Ethiopian Population and Housing Census conducted by the Central Statistical Agency in 2007 was used as a sampling frame. A complete list of 84,915 Enumeration Areas (EAs) was used as a sampling frame to select the EAs for EDHS 2016, and each EA comprised 181 households. A two-stage stratified cluster sampling technique was used to conduct the survey. The regions were stratified into urban and rural, producing 21 strata. In each stratum, sample EAs were selected independently in two stages using proportional allocation and implicit stratification. Based on the 2007 Population and Housing Census, 645 EAs (202 in urban areas and 443 in rural areas) were selected in the first stage. In the second selection stage, 28 households per cluster were chosen with an equal probability of systematic selection supported by the newly created household listing.

Further detailed information about the sampling procedures and household selection is provided in the 2016 EDHS report (46). In this study, the individual characteristics of the respondents aged 15–24 years listed in the 2016 EDHS were used. A sample size of 10,594 youth was used for the final analysis from 645 EAs.

### Study variables

The outcome variable of this study was self-reported. In the EDHS, questions were asked about substance use. The participants were asked four questions about whether they currently smoke cigarettes, pipes, or any other tobacco products, which were to be answered with a “yes” or “no.” The youth were classified as “cigarettes or tobacco smokers” if the response was “yes.” Again, two questions regarding alcohol drinking and Khat chewing were asked—“during the last 30 days preceding the survey, how many days the participants have a drink that contains alcohol, and how many days the participants have chew Khat, respectively?” Based on these questions, the youth were classified as “people who drink alcohol” and “people who chew Khat” if the response was “one or more days” (including occasionally or daily). Those who have no history of using those substances were considered “non-users.” There are also questions to be answered by “yes” or “no” about whether currently they use marijuana and shisha or nothing. However, there are no such cases reported in the survey. Shisha is a psychoactive substance that contains nicotine and psychoactive ingredients like cigarettes.

As a result, we have not included marijuana and shisha use in the analysis. Finally, for the simplicity of analysis, substance use was considered using specified substances such as alcohol, Khat, and cigarette/tobacco smoking, giving a sum-total score ranging from zero to three. Then, the sum score of the specified substances was categorized as “yes” if the total score was greater than zero and “no” if the sum score was zero. Therefore, in this study, substance use constitutes occasional or daily use of at least one of the specified substances such as alcohol, Khat, and/or tobacco within 30 days preceding the survey. The DHS contains no information about the standard amount of use of the products and duration of service in a session. Therefore, it was difficult to estimate the binge users.

According to the WHO, youth in the present study denotes late adolescents and young adults aged between 15 and 24 years (47). The individual- and community-level variables were considered independent variables in the study. Sex, age, marital status, educational achievement, household wealth index, individual media exposure, occupation status, and religion were the individual-level factors. Some of these factors were recategorized for the simplicity of analysis. Peer pressure, the presence of a family member who uses substances, and other reasons for using substances were not examined in this study and, therefore, were not included in the analysis. Place of residence, region, and community-level media exposure were considered community-level factors. In the EDHS, participants' media exposure was ascertained by three survey questions to be answered “not at all,” “at least once a week,” and “more than once a week” for the questions “how often do you have read newspaper or magazine; how often do you have listening radio, and how often do you have watching television?” Based on these questions, the individual level of media exposure was obtained by aggregating the specified ways of getting information, such as reading news or magazine, listening to the radio, and watching television which gives a sum-total score ranging from zero to six. Then, the total score of media exposure was categorized as “yes” if the total score was greater than zero and “no” if the sum score was zero. Therefore, in this study, an individual's media exposure was defined as those who have a chance to get information through at least one of the three specified mass media such as reading news or magazines, listening to the radio, and/or watching television at least once per a week.

The community-level media exposure was obtained by aggregating the individual-level media exposure into groups using those who had media exposure. This community-level media exposure shows the general media exposure within the community. Since the aggregated variable had a skewed distribution, the median values were categorized as higher and lower. This study region was recategorized into three categories; larger central [Tigray, Amhara, Oromia, and Southern Nations Nationalities and People's Region], small peripherals [Afar, Somali, Benishangul, and Gambela], and metropolis [Harari, Dire Dawa, and Addis Ababa] supported by their geopolitical features, according to previous studies from Ethiopia (48, 49).

### Data management and statistical analysis

The extracted EDHS data included the youth respondents' sociodemographic and behavioral characteristics. The cleaned and recoded data were analyzed using STATA version 14. Descriptive statistics such as frequencies and percentages of variables were presented using texts and tables. Sample weights were performed throughout the analysis to restore the representativeness and to adjust the nonproportional allocation of the sample to enumeration areas (clusters) and regions during the survey process. A mixed multilevel logistic regression analysis was employed to account for the hierarchal nature of the EDHS data. First, a bivariable multilevel logistic regression analysis was performed, and those variables with a *p*-value of < 0.20 were selected for multivariable analysis. In the multivariable analysis, variables with a *p*-value of < 0.05 were considered statistically significant, and the factors associated with substance use were reported by an adjusted odds ratio (AOR) at a 95% confidence interval.

After selecting variables for multivariable analysis, four models; the null model (without explanatory variables), model II (containing only individual-level factors), model III (examined the effect of community-level factors), and model IV (which constitutes both individual and community-level factors) were fitted. Model comparison and fitness were assessed using the deviance and Akaike information criterion (AIC), and the model with lower deviance and AIC (Model IV) was selected as the best-fitted model. However, it can be hard to interpret the AOR in multilevel logistic regression since the community-level variables are constant for all individuals in the clusters. A better way of interpreting the AOR is by contrasting two clusters differing in the value of the contextual variable by one unit. In the final model (model IV), we have used the 80% interval odds ratio (IOR-80%) to address this limitation in interpreting the effects of community-level variables. It quantified the AOR using the community variance. The IOR-80% is defined as the middle 80% range of the distribution of OR formed by making the random pairwise comparison between the clusters exposed and nonexposed to the contextual variable. The IOR-80% interval is narrow if the variation of substance use between clusters $\delta_{\text{u}}^{2}$ is small and is comprehensive if the substance use variation between clusters is considerable. For some clusters, the association is opposite to the AOR if the IOR-80% interval is 1 (50, 51).

In addition, the measures of community variation (random effects), which are the measures of variation of substance use across communities or clusters, were estimated by the intra-class correlation (ICC), median odds ratio (MOR), and proportional change in variance (PCV) (51–53). These ICC, MOR, and PCV values were calculated to quantify the degree of homogeneity of substance use within clusters, the degree of variation of substance use across clusters in terms of the odds ratio scale, and the proportion of variance explained by consecutive models, respectively.

## Results

### Descriptive statistics of the youth characteristics

Weighted samples of 10,594 youth aged 15 to 24 years were included in this data analysis. More than half of the participants (57.98%) were female and 43.86% were Orthodox Christians. Nearly two-thirds of the participants were never married, 57.35% of them had attended primary school, and 59.78% of the participants were employed or had private work. The prevalence of alcohol drinking was higher among the youth living in the Amhara region (77.73%) and Khat chewing was more prevalent among individuals living in rural areas (13.99%) than those living in urban areas. Moreover, drinking alcohol, chewing Khat, and smoking were more prevalent among the youth aged 20 to 24 years than those aged 15 to 19 years. Higher proportions of individuals with lower monthly income were Khat chewers, but alcohol use was higher among individuals with higher monthly income (Table 1).

**Table 1:** Descriptive statistics of substance use by the youth according to the 2016 EDHS data (weighted data = 10,594).

**Variables**	**Categories**	**Weighted sample (%)**	**Youth who consume at least a specified substance, %**	**Youth who drinks any alcohol, %**	**Youth who chew Khat, %**	**Youth who smokes any tobacco products, %**
Sex	Male	4,451 (42.02)	56.26	45.20	18.05	1.69
	Female	6,143 (57.98)	40.00	31.09	8.58	0.43
	*p*-value		< 0.001	< 0.001	< 0.001	< 0.001
Age	15–19 years	5,952 (56.19)	43.06	34.18	10.16	0.27
	20–24 years	4,642 (43.81)	51.66	39.11	15.64	1.83
	*p*-value		< 0.001	< 0.001	< 0.001	< 0.001
Religion	Orthodox Christians	4,647 (43.86)	75.97	75.25	3.16	0.52
	Muslim	3,218 (30.38)	36.96	4.29	34.88	2.05
	Protestant	2,500 (23.60)	6.92	6.61	1.68	0.34
	Others^*^	229 (2.19)	29.88	21.92	8.49	0.94
	*p*-value		< 0.001	< 0.001	< 0.001	< 0.001
Marital status	Never married	7,387 (69.73)	45.70	36.98	10.81	0.68
	Married	2,796 (26.39)	47.19	32.10	17.05	1.61
	Others^**^	411 (3.88)	64.72	53.72	13.41	1.35
	*p*-value		< 0.001	< 0.001	0.003	< 0.001
Educational attainment	No formal education	1,771 (16.71)	50.94	35.22	17.32	1.13
	Primary school	6,076 (57.35)	44.87	33.21	13.48	0.88
	Secondary school	2,094 (19.77)	47.00	42.09	7.47	0.91
	Higher	653 (6.17)	53.36	50.11	7.43	1.27
	*p*-value		< 0.001	< 0.001	0.003	0.103
Occupation status	Not working	4,261 (40.22)	34.63	26.19	9.33	0.74
	Have work	6,333 (59.78)	55.04	43.17	14.73	1.09
	*p*-value		< 0.001	< 0.001	< 0.001	0.026
Media exposure	Yes	6,038 (56.99)	51.06	42.61	11.35	1.00
	No	4,556 (43.01)	41.22	28.03	14.16	0.89
	*p*-value		< 0.001	< 0.001	0.047	0.068
Household Wealth index	Poorest	1,614 (15.24)	45.47	29.95	16.62	1.02
	Poorer	1,835 (17.32)	49.47	33.57	18.32	1.45
	Middle	1,960 (18.50)	48.93	35.88	14.45	1.01
	Richer	2,306 (21.76)	44.98	36.81	9.80	0.60
	Richest	2,879 (27.18)	45.96	41.63	7.53	0.84
	*p*-value		0.063	< 0.001	< 0.001	0.003
Region	Tigray	808	76.59	75.94	1.73	0.41
	Afar	84	14.27	6.93	7.32	2.62
	Amhara	2,510	82.76	77.73	7.15	0.32
	Oromo	3,886	36.01	14.89	23.11	1.29
	Somalia	310	9.60	0.65	9.30	3.00
	Benishangul-Gumz	108	38.64	32.32	8.78	2.95
	SNNP	2,167	18.07	14.29	5.39	0.54
	Gambella	33	34.22	31.10	8.98	2.11
	Harari	26	36.02	8.94	29.70	2.05
	Addis Ababa	598	57.79	55.35	8.45	1.74
	Dire-Dawa	64	38.41	16.96	26.08	2.35
	*p*-value		< 0.001	0.004	< 0.001	< 0.001
Regional class	Large central	9,372 (88.46)	47.88	36.85	12.89	0.78
	Small peripheral	535 (5.05)	17.72	9.91	8.87	2.88
	Metropolitan	687 (6.49)	55.17	50.03	10.89	1.81
	*p*-value		< 0.001	< 0.001	< 0.001	< 0.001
Living Residence	Rural	8,260 (77.97)	47.23	34.92	13.99	0.97
	Urban	2,334 (22.03)	45.42	41.35	7.51	0.88
	*p*-value		0.175	< 0.001	0.031	0.683
Community media exposure	Lower	5,843 (55.15)	44.45	31.93	13.59	0.93
	Higher	4,751 (44.85)	49.75	41.77	11.30	0.98
	*p*-value		< 0.001	< 0.001	0.243	0.679

Others^*^: Catholic, traditional; Others^**^: divorced, widowed, separated.

#### The prevalence of substance use

In 2016, 46.74%, with a 95% CI of 45.88 to 47.78, of Ethiopian youth aged 15 to 24 years (56.26% male subjects and 40% female subjects) reported consuming at least one specified substance (alcohol, Khat, or cigarette/ tobacco products). Of the participants, 36.34% (*n* = 3,850), 12.56% (*n* = 1,331), and 0.95% (101) were drinking alcohol, chewing Khat, and smoking any tobacco products, respectively. Of those who reported using these specified substances, 45.2% of male subjects and 31.09% of female subjects reported drinking alcohol and 18.05% and 1.69% of male subjects were chewing Khat and smoking any tobacco products, respectively (Table 2).

**Table 2:** The prevalence of substance use in Ethiopia, 2016 (weighted data).

	**Total, %**	**Male, %**	**Female, %**
Specified substance users (alcohol, Khat, or cigarette/other tobacco products)	46.74	56.26	40.00
Alcohol drinkers	36.34	45.20	31.09
Khat chewers	12.56	18.05	8.58
Cigarettes/other tobacco product smokers	0.95	1.69	0.43

Substances using defined as the use of at least one of the specified substances (alcohol, Khat, and tobacco) occasionally or daily in the preceding 30 days of the survey.

### Random effects and model fitness

In the null model (model I), there was a significant variation in the log odds of people who use substances across the communities (σ2 u0 = 3.36, *P* < 0.001, and 95%CI: 2.85–3.96). This variation remained significant after controlling all models' individual- and community-level factors. As shown by the estimated ICC coefficient, about 50.53% of the total variation of the people who use substances could be attributed to the cluster-level effects (unexplained variation). The null model also had the highest MOR value (5.71), indicating that when randomly selecting an individual from one cluster with a higher risk of substance use and the other cluster at lower risk, individuals in the cluster with a higher risk of substance use had 5.71 times higher odds of having used at least one substance (alcohol, Khat, or smoking/any tobacco products) as compared to their counterparts. In addition, the highest PCV (48.8%) in the full model (model IV) indicates that the log odds of having substance use variation across the community level, and the 48.8% of the variation was explained by the combined factors at both the individual and community levels (Table 3).

**Table 3:** Model comparison and goodness-of-fit test in the multilevel analysis.

**Random effect**	**Model I**	**Model II**	**Model III**	**Model IV**
Community variation (SE)	3.36^**^ (0.28)	2.64^**^ (0.31)	2.14^**^ (0.27)	1.72^**^ (0.23)
ICC	0.5053	0.4452	0.3941	0.3433
MOR	5.71	4.68	4.01	3.48
PCV	Ref	0.214	0.363	0.488
**Model fit statistics**	**Model I**	**Model II**	**Model III**	**Model IV**
Log-likelihood ratio test	−5248.36	−4916.29	−4880.34	−4560.45
Deviance	10496.72	9832.58	9760.68	9120.90
AIC	10500.72	9862.58	9782.68	9158.91

^**^p < 0.001.

The model fitness was ascertained using the log-likelihood, deviance, and AIC values as indicated in Table 3, in which lower values were observed in the full model (model IV). This indicates that model IV for youth substance use was a better explanatory model. This also suggests that the addition of the community compositional factors increased the ability of the multilevel model, indicating the goodness-of-fit of the multilevel model.

### Factors associated with substance use

The bi-variable multilevel modeling showed that sex, age, marital status, educational attainment, household wealth index, occupational status, individual's media exposure, living residence, living region, and community-level media exposure have a *p*-value of < 0.2. A multivariable multilevel logistic regression analysis, where the individual- and community-level factors were fitted simultaneously, indicated that male sex, 20–24 years of age, having a job, exposure to media, and living in large central and metropolitan regions were significantly associated with substance use with a *p*-value of < 0.05.

The odds of substance use among the youth were 2.65 times [AOR = 2.65; 95% CI: 2.12, 3.30] higher in male subjects than female subjects. The odds of substance use were 57% [AOR = 1.57; 95%CI; 1.31, 1.88] higher in the youth in the age group of 20–24 years compared with the youth in the age group of 15–19 years. Regarding individual media exposure, youth who had exposure to media at the individual level had 1.49 times [AOR= 1.49; 95% CI: 1.17, 1.90] higher odds of substance use as compared to those who have not been exposed to mass media (reading news and/ magazine; listening radio, and/ watching television). The youth who had a job had 1.68 times [AOR = 1.68; 95% CI: 1.39, 2.03] higher odds of substance use as compared with the youth who have not worked. The 80% IOR value for the living region characteristics in model IV were (2.06, 239.13) and (1.29, 149.46) for large central regions vs. small peripheral regions and metropolitan regions vs. small peripheral regions, respectively. They were (0.22, 24.95) for urban residence vs. rural residence and (0.26, 30.18) for the presence of higher vs. lower community-level media exposure. Thus, when comparing the youth with identical characteristics, one selected from a large central region or a metropolitan region and one from a small peripheral region, the odds of substance use will lie between (2.06 and 239.13) or (1.29 and 149.46), respectively, in 80% of such comparisons (Table 4).

**Table 4:** Multivariable multilevel logistic regression results of both individual- and community-level factors associated with substance use in youth, EDHS 2016.

**Variables**	**Model I**	**Model II (AOR 95% CI)**	**Model III (AOR 95% CI)**	**Model IV (AOR 95% CI)**
**Individual-level factors**				
**Sex**				
Female		1.00		1.00
Male		2.62^***^ (2.10, 3.25)		**2.65** ^**^ **(2.12, 3.30)**
**Age**				
15–19 years		1.00		1.00
20–24 years		1.58^***^ (1.32, 1.88)		**1.57** ^**^ **(1.31, 1.88)**
**Educational attainment**				
Higher		1.00		1.00
Secondary school		0.92 (0.58, 1.44)		0.91 (0.58, 1.43)
Primary school		0.95 (0.61, 1.48)		0.93 (0.60, 1.46)
No education		0.84 (0.52, 1.34)		0.84 (0.52, 1.35)
**Marital status**				
Married		1.00		1.00
Never married		0.81 (0.64, 1.02)		0.80 (0.63, 1.01)
Others^*^		1.06 (0.69, 1.62)		1.04 (0.68, 1.59)
**Occupational status**				
No work		1.00		1.00
Have Job		1.71^**^ (1.41, 2.07)		1.68 (1.39, 2.03)
**Wealth Index**				
Poorest		1.00		1.00
Poorer		1.09 (0.81, 1.46)		0.98 (0.73, 1.33)
Middle		1.20 (0.89, 1.62)		1.08 (0.79, 1.46)
Richer		1.09 (0.79, 1.51)		0.97 (0.70, 1.35)
Richest		1.35 (0.96, 1.90)		1.25 (0.84, 1.86)
**Media exposure**				
No		1.00		1.00
Yes		1.54^**^ (1.27, 1.85)		**1.49** ^*^ **(1.17, 1.90)**
**Community-level variables**				
**Living Residence**				
Rural			1.00	1.00
Urban			0.87 (0.60, 1.27)	0.84 (0.51, 1.40)
80% IOR				0.22, 24.95
**Living region**				
Small Peripheral			1.00	1.00
Large central			6.99^***^ (4.85, 10.07)	**3.10** ^***^ **(1.84, 6.40)**
80% IOR			5.40^**^ (3.67, 7.94)	(2.06, 239.13)
Metropolitan				**2.63** ^**^ **(1.37, 5.39)**
80% IOR				1.29, 149.46
**Community media exposure**				
Lower			1.00	1.00
Higher			1.69^**^ (1.41, 2.03)	1.03 (0.80, 1.34)
80% IOR				0.26, 30.18

^*^p-value < 0.05; ^**^p-value < 0.01; ^***^p-value < 0.001. The bold values indicate statistically significant.

## Discussion

The youth is the most turbulent time in human development and is highly prone to multiple risk-taking behaviors. Alcohol, Khat, and tobacco are the common substances used by Ethiopian youth. This study attempted to assess the prevalence and determinant factors of substance use among youth in Ethiopia.

In this study, the prevalence of substance use among the youth in Ethiopia was 46.74%, with a 95% CI of 45.88–47.78. This finding was in line with the reports in sub-Saharan Africa (40–59.9%) (54) and Nigeria (46.3%) (55). Conversely, our finding showed a lower prevalence than that reported in other studies conducted in Ethiopia (30), Eastern Africa (29), Rwanda (56), South Africa (57), and Nigeria (58). On the other hand, the result of this study showed a higher prevalence than that reported in previous studies conducted in Rwanda (59) and Egypt (60). The variation might be due to the study area and sample size differences as most of the indicated studies were institution-based with a small sample size in which lower rates of substance consumption are expected compared to this large population-based study. The discrepancies could also be due to the differences in the study population, study design, and interest to measure. For example, small geographical, institution-based, and standardized substance consumption identification questionnaires were used in most of these studies, while national-level population-based data and substance use behavior assessment DHS questions were used in this study. When data are collected from a single institution, a higher/lower rate of individuals may consume substances if there is higher/lower availability and use pattern of drugs in the community. A systematic review and meta-analysis study among in-school youth was used in an eastern African study whereas a cross-sectional study among individuals aged 15–24 years from the 2016 EDHS data was used in this study. In addition, the interest of study was on fewer substances in a Nigerian study among university students. Instead, we have included alcohol, Khat, and tobacco products which are commonly used in Ethiopia. Therefore, variations in the magnitude of multiple substances vs. fewer substances are expected. Besides, the discrepancies between the finding of this study and that of the findings of studies conducted in Ethiopia might be due to sociodemographic and cultural differences. This is due to the fact that consuming substances like alcohol, Khat, and tobacco products differ across countries and subpopulations.

One unexpected finding in this study is that no one from the sample population has admitted to the recent use of cannabis and shisha. This finding is different from previous studies in which there was a high prevalence of cannabis and shisha use in the Ethiopian community (61, 62). The possible reason might be that previous studies were conducted in a specific area where the products are produced and available in the community. Cannabis is frequently consumed in Shashemane town, southern Ethiopia where the previous studies were conducted. Most of the factors associated with substance use in this study were different from those reported in previous studies in Ethiopia. For example, living in large central and metropolitan regions, individuals' media exposure and having work (job) were not addressed and associated in the other studies. In this study, we observed that male youth were more likely to use substances compared to female youth. This report is consistent with those of other studies (63–65), which showed that male youth commonly use substances. The possible justification for male youth having higher odds of substance use was undesirable masculine traits, like drinking, chewing, or smoking to reduce distress and a tendency to bypass social sanctions (66). The other explanation for the gender difference might be substances like alcohol, Khat, and tobacco are commonly practiced and socially regarded as a male habit in Ethiopian culture.

The age of the youth is an important contributor to substance use in which the odds of substance use were higher among older youth aged 20–24 years compared to younger youth aged 15–19 years. This finding was in agreement with other studies done in Ethiopia (63, 65), Rwanda (59), and Nigeria (58). This could be due to the fact that the use of substances among youth almost always increases as their age increases (67). Furthermore, the younger population has a higher rate of substance use, with adolescence being the critical period for initiation and the young age (18–25 years) being the peak age for substance use (11, 12). This study revealed that the odds of substance use were higher among the youth who had a job compared to those who have not worked. This higher proportion may likely be related to the adequate income needed to buy the substances. The higher the income, the higher the chances of abusing drugs by the youth. As observed from the result, most Khat chewers are individuals with lower monthly incomes as Khat can be easily cultivated on their agricultural land and is cheaper to buy. At the same time, alcoholism was higher among individuals with higher monthly incomes as more money is required to buy alcohol than that is needed to buy Khat.

In this study, the youth who had media exposure (reading newsletters/magazines, listening to the radio, and/or watching television) at the individual level were more likely to consume substances than those who have no media exposure. This finding is in agreement with another study (35). This could be because the youth populations are vulnerable to substance use, which is encouraged by the deception and manipulation strategy of substance advertising and marketing (41). This implies that banning drug advertisements using mass media, controlling alcohol, Khat, and tobacco product marketing for under ages, and expanding substance use–related education using mass media are essential to intervene in youth before and after starting substance use.

Furthermore, this study region is also associated with youth substance use. The youth from large central and metropolitan regions were more likely to have substance use as compared to small peripherals. This is consistent with a study done in Rwanda (56), India (68), and England (69), which showed that regional variation is a consistent predictor of substance use. This might be due to the easy availability and access to big commercial trade with one another and higher challenges of life due to lower social relationships in urban areas like metropolitan regions (70). In addition, large central regions are more agrarian regions where the survivors have produced local alcoholic beverages, such as “Tella,” “Areki,” and “Tej” in the rural areas and “beer” and “wine” in the urban areas, from agricultural products than in the small peripherals (71).

One of the main limitations of this study was that the DHS did not have information about the standard amount of use of substances and duration of use in a session which limited us from identifying the binge use of the products in the survey. Second, underreporting of substance-use behavior is common because of social desirability bias as consuming substances may be perceived as socially and culturally undesirable, making it difficult to determine the accurate magnitude of substance use through the survey. Due to logistical reasons, the researchers might have experienced difficulties in reaching locals. Therefore, to overcome such difficulties, they might have collected data from participants who could be reached easily. This sampling bias could influence the representativeness of the findings. Another limitation of this work might be the recall bias because the data collection took place 6 years before the survey of the study. Besides, the research could not show the cause–effect relationships between factors and outcomes owing to its cross-sectional nature.

## Conclusion

The prevalence of using at least one substance in the youth in Ethiopia was high. Male sex, 20–24 years of age, exposure to media, having a work/job, and living in large central and metropolitan regions were the factors associated with the problem. It is crucial that the policymakers strengthen the application of the existing policies, especially controlling alcohol, Khat, and tobacco product marketing for minors, smoking in public places, and banning broadcasting alcohol advertisements for reducing the prevalence and consequences of substance use among individuals aged 15–24 years. As expressed in the introduction, the cost and excise tax of the products in our country are lower compared to other countries. Therefore, increasing the retail cost and excise tax of alcohol and tobacco products might help in reducing the number of users and the risky consumption of substances. Yet again, special interventions targeting risky youth such as those living in large central and metropolitan areas are also needed to intervene in substance use at an early age.

## Data availability statement

The original contributions presented in the study are included in the article/supplementary materials, further inquiries can be directed to the corresponding author.

## Ethics statement

Ethical review and approval was not required for the study on human participants in accordance with the local legislation and institutional requirements. The patients/participants provided their written informed consent to participate in this study. Written informed consent was obtained from the individual(s) for the publication of any potentially identifiable images or data included in this article.

## Author contributions

TK and BT developed the proposal, requested the data from DHS, extracted, recoded, analyzed the data, and wrote the draft manuscript. GT assisted in proposal development, data analysis, and manuscript writing. TA extracted, cleaned, and recoded the data and checked the data analysis. SK revised the proposal and assisted with manuscript writing. BL revised the proposal and revised and approved the manuscript. Finally, all authors have read and approved the manuscript.
